# Hybridization and diversity of aquatic macrophyte *Sparganium* L. (Typhaceae) as revealed by high-throughput nrDNA sequencing

**DOI:** 10.1038/s41598-022-25954-0

**Published:** 2022-12-14

**Authors:** Evgeny A. Belyakov, Yulia V. Mikhaylova, Eduard M. Machs, Peter M. Zhurbenko, Aleksandr V. Rodionov

**Affiliations:** 1 Papanin Institute for Biology of Inland Waters, Russian Academy of Sciences, Yaroslavl Region, Nekouz District, 109, Borok, Russia 152742; 2 Cherepovets State University, Lunacharsky Ave., 5, Cherepovets, Russia 162600; 3 Komarov Botanical Institute, Russian Academy of Sciences, Prof. Popova St., 2, St. Petersburg, Russia 199376; 4 St. Petersburg State University, Universitetskaya Embankment, 7-9, St. Petersburg, Russia 199034

**Keywords:** Evolutionary genetics, Molecular evolution, Speciation, Taxonomy, Evolutionary biology, Plant evolution, Plant hybridization, DNA sequencing, Next-generation sequencing

## Abstract

*Sparganium* is an emergent aquatic macrophyte widely spread in temperate and subtropical zones. Taxa of this genus feature high phenotypic plasticity and can produce interspecific hybrids. By means of high-throughput sequencing of the internal transcribed spacer (ITS1) of 35S rDNA, the status of 15 Eurasian *Sparganium* species and subspecies was clarified and the role of hybridization events in the recent evolution of the genus was investigated. It has been shown that a number of species such as *S. angustifolium, S. fallax* and *S. subglobosum* have homogenized rDNA represented by one major ribotype. The rDNA of other taxa is represented by two or more major ribotypes. Species with high rDNA heterogeneity are apparently of hybrid origin. Based on the differences in rDNA patterns, intraspecific diversity was identified in *S. probatovae* and *S. emersum*. Thus, we have concluded that *Sparganium* has extensive interspecific hybridization at the subgenus level, and there may also be occasional hybridization between species from different subgenera.

## Introduction

*Sparganium* L. (Typhaceae) are aquatic plants widely spread in temperate and subtropical zones of the Northern and Southern Hemispheres^1,2^. *Sparganium* species represent an important part of freshwater vegetation. They serve as edificator plants and influence the geomorphology of river courses^3,4^.

The genus *Sparganium* is divided into two subgenera*, Sparganium* (or *Melanosparganium*) and *Xanthosparganium* Holmb*.*^1,5^, and three sections^6^. The subgenus *Sparganium* includes the section *Erecta* Aschers. et. Graebner*,* and the subgenus *Xanthosparganium* includes sections *Natantia* Aschers. et. Graebner and *Minima* Aschers. et. Graebner^2,5,6^*.*

The number of species and subspecies within the genus *Sparganium* and their taxonomic rank is the subject of longstanding scientific discussions^1,2,7–9^. Researchers estimate the number of *Sparganium* species as between 15 and 20^2,6^. Similar to a number of other aquatic genera, some *Sparganium* species and subspecies lack clear morphological differences^1,5,7,9–12^. For this reason, some researchers consider the morphotype/group of certain natural populations in the rank of species, while others assign them to the rank of subspecies or forms only. For example, the taxa *S. emersum* Rehm*.* (Eurasia and western North America), *S. acaule* (Beeby ex Macoun) Rydb. (eastern North America) and *S. rothertii* Tzvelev (Eurasia) can be considered as individual species^12,13^ or subspecies of the polymorphic species *S. emersum* s.l.^1,14^. Another example is the taxa *S. neglectum* Beeby and *S. microcarpum* (Neum.) Domin; they can be treated as a different species^5^ or subspecies of *S. erectum* L.^9,11^.

Intermediate morphological forms are very common in *Sparganium*. It appears that reproductive isolation between species in the genus is not complete and allows interspecific hybridization^1,2,9,15–17^. *Sparganium* hybrids can be sterile, partially fertile, or fertile^1,17–19^. The existence of seven hybrid taxa in the genus *Sparganium*^2,9,12,17,20^ has now been proved by molecular analysis methods. However, the importance and distribution of interspecific hybridization in the speciation of *Sparganium* have been poorly studied so far.

A valuable tool for detecting plant hybrids in nature is the study of intragenomic rDNA polymorphism^21,22^. rDNA fragments are the most commonly used molecular markers in plant phylogeny^23,24^ thanks to their unique properties. 35S rDNA contains both coding conserved fragments (18S, 5,8S, and 26S rRNA genes) and noncoding highly variable fragments (internal transcribed spacers ITS1 and ITS2). Plants contain from 200 to 22,000 35S rDNA cistrons per haploid genome^25^ arranged as long tandem repeats on one or more chromosomes^26^.

First-generation interspecific hybrids always have both parents' rDNA in the genome^27–30^. In the next generations, some species retain rDNA^31–33^, while others undergo rDNA homogenization, resulting in the loss of most of the rDNA of one of the parents and only a minor number of 35S rDNA loci remaining in the genome, which can be detected via sequencing cloned PCR products^22,28,33–35^ or by using next-generation-sequencing (NGS)^16,21,36–41^. Analysis of the intraindividual polymorphism of 35S rDNA fragments has been successfully used to study hybrids in various groups of plants^29–33^. ITS1 and ITS2 fragments are most variable at the intraindividual level^42^. We chose the ITS1 fragment of the 35S rDNA^43^ locus as a marker, because it was shown that homogenization was usually lower in ITS1 than in ITS2, suggesting that concerted evolution operates less efficiently on the former^44^.

The main objective of this study was to investigate the role of hybridization events in the recent evolution of *Sparganium*. We applied the NGS method to analyze the intragenomic polymorphism of the 35S rRNA gene ITS1 region. Fifteen *Sparganium* taxa were studied, including both morphologically well-identified species and subspecies, some of likely hybrid origin, as well as plant specimens that exhibited morphological traits intermediate between the two species (i.e., probable hybrids). We set out to address the following questions: (1) Does the molecular analysis prove the hybrid origin of morphologically intermediate forms identified on the basis of the morphological analysis? (2) Can well-identified species have characters of hybrid origin in their genomes? (3) Is the hypothesis of hybrid origin of *S. probatovae* Tzvelev and *S. rothertii* put forward by Tsvelev^13^ validated? (4) Is hybridization between species from different sections and species from different subgenera possible in the genus *Sparganium*?

## Results

### Identification and phylogenetic analysis of the major ribotypes

To assess the intragenomic diversity of *Sparganium* species rDNA, we analyzed ITS1 polymorphism in 37 samples belonging to 15 species and subspecies using Illumina high-throughput sequencing. Four species and subspecies from section *Erecta*, two species from section *Minima*, and nine species from section *Natantia* were studied. The USERCH^45^, and UNOISE^46^ algorithms were employed to process Illumina high-throughput sequencing data to identify the unique variants of the ITS1 sequences flanked by 18S rDNA and 5.8S rDNA as well as to calculate their comparative frequency (number of read pairs uniquely mapped to one variant). Obtained denoised sequences are called ZOTUs (zero-radius OTUs)^46^, or ribotypes^21,39,40^. ZOTUs with a read count of 10 or more were used in the further analysis. In our study, we divided ribotypes into major ones, which accounted for at least 2% of the reads in the sample, and minor ones. A total of 64 major ribotypes and about 150 minor ribotypes were identified. Data on the occurrence and abundance of each ribotype in the studied plants are presented in Supplementary Table S1, with each major ribotype given a unique name. Some major ribotypes differed from each other by 1–2 SNPs; we designated these variants by letters, combining them into families (e.g., Negle-3A & Negle-3B). The table of SNPs and indels (Supplementary Table S2) shows each family of these variants as a single line. The data for each family are summarized in the graphs (Fig. 2, 3, 4). Some sequences have large indels; for example, Roth-1 and Long-3 have a 70 bp deletion, Negle-4 has a 13-nucleotide deletion, and the sequences Hyper-1 and Hyper-2 contain 3-nucleotide CCC insertions (Supplementary Table S2).

**Figure 1:**
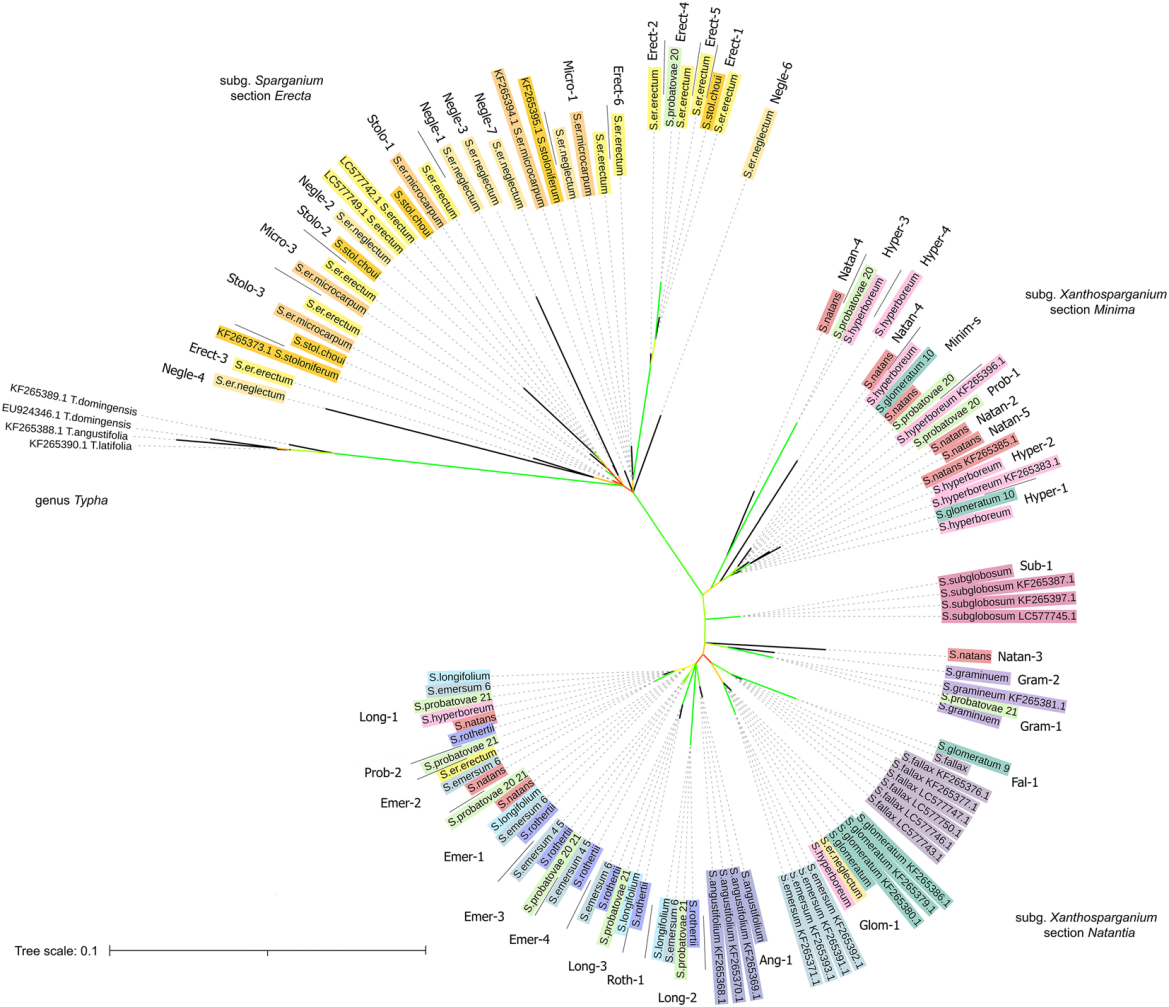
Phylogenetic tree of *Sparganium* major ribotypes built by maximum likelihood method. Bootstrap values: green: 80–100, light-green: 60–80, yellow: 40–60, orange: 25–40.

Major ribotypes along with NCBI Sanger sequences of species of *Sparganium* and *Typha* genera were used to perform phylogenetic analysis (Fig. 1). In the phylogenetic tree, most of the major ribotypes of each section (Supplementary Table S1 and S2) are clustered together, forming three clades. ITS1 sequences of the subgenus *Sparganium* appear to be placed closer to the genus *Typha*, thus occupying a basal position in the genus *Sparganium*. The longest branch separates two subgenera, *Sparganium* and *Xanthosparganium*, and has high bootstrap value. In the subgenus *Xanthosparganium*, the major ribotypes of section *Minima* also form one clade with a high bootstrap value. The major ribotypes of section *Natantia* are clustered together but it is difficult to obtain accurate phylogenetic relationships between them due to low bootstrap support.

It is worth noting that some species have species-specific ribotypes that are related to the ribotypes of other sections. For example, "Natan-3" ribotype found in *S. natans* (section *Minima*) cluster with ribotypes of section *Natantia*, and "Prob-1" ribotype found in *S. probatovae* (section *Natantia*) clusters with ribotypes of section *Minima*.

### Number of major ribotypes across species

**Figure 2:**
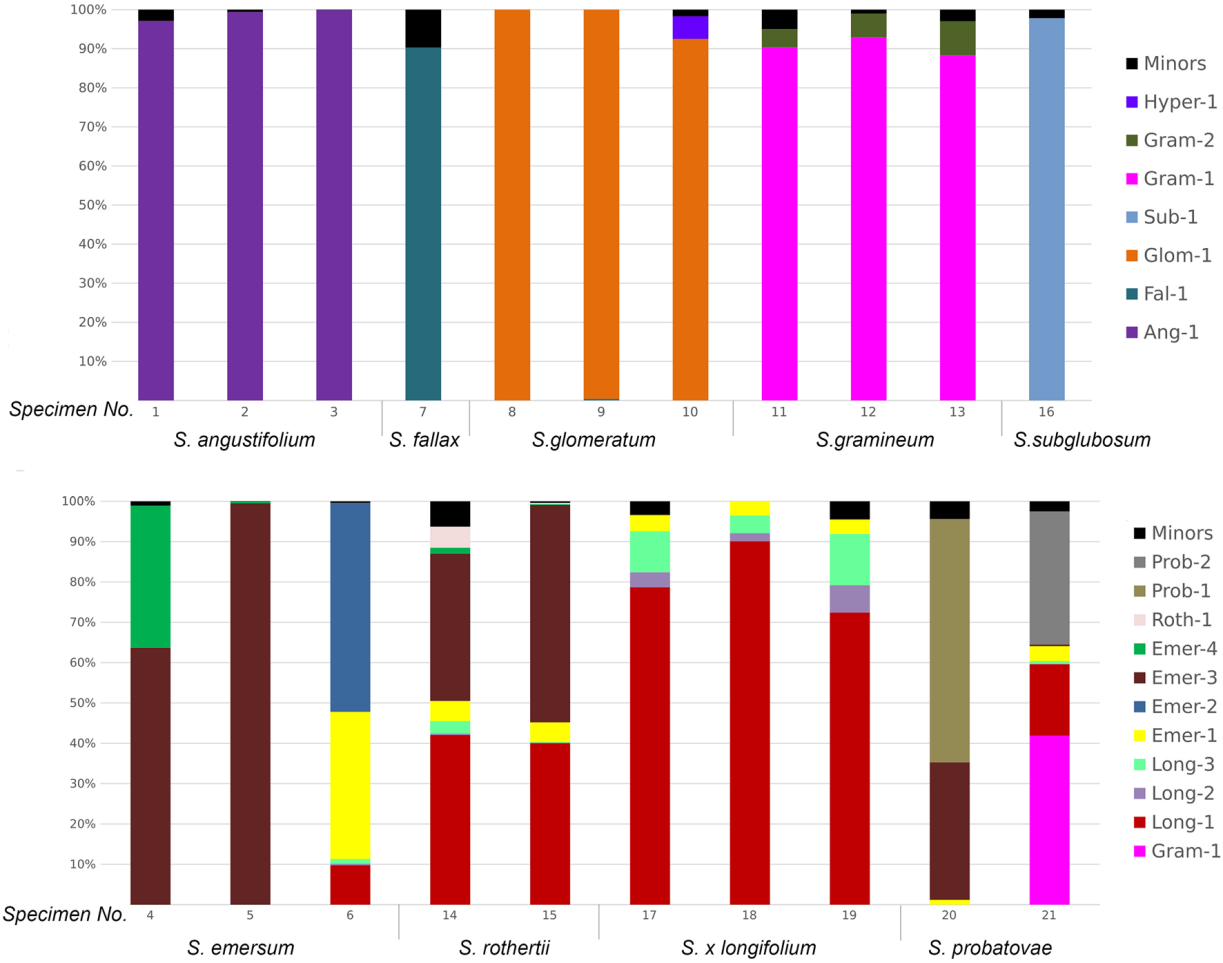
Stacked bar plots showing the relative abundance of the major ribotypes across the species of subgenus *Xanthosparganium* section *Natantia*. Minor ribotypes in a sample are collectively termed "Minors".

**Figure 3:**
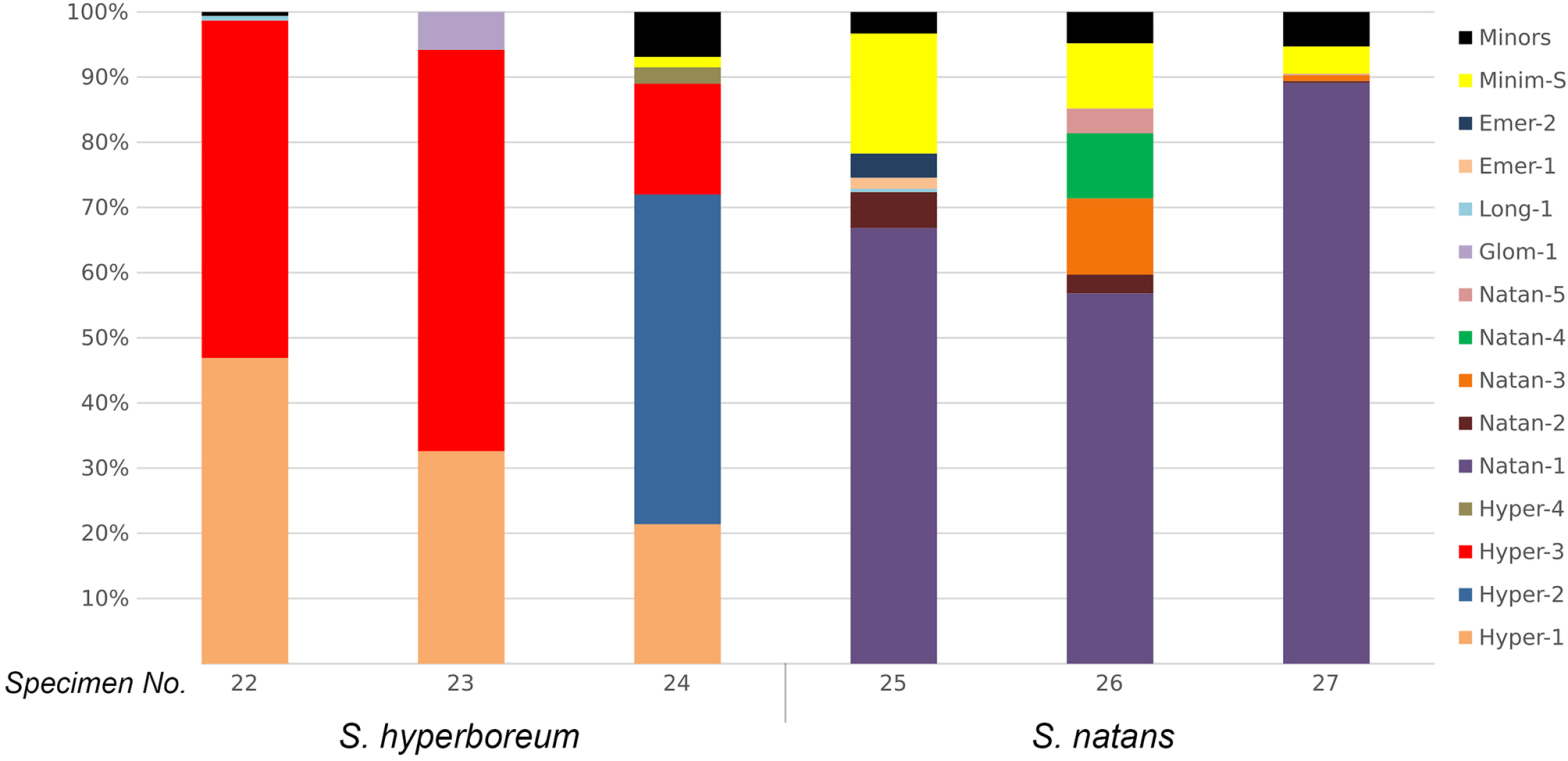
Stacked bar plots showing the relative abundance of the major ribotypes across the species of subgenus *Xanthosparganium* section *Minima*. Minor ribotypes in a sample are collectively termed "Minors".

**Figure 4:**
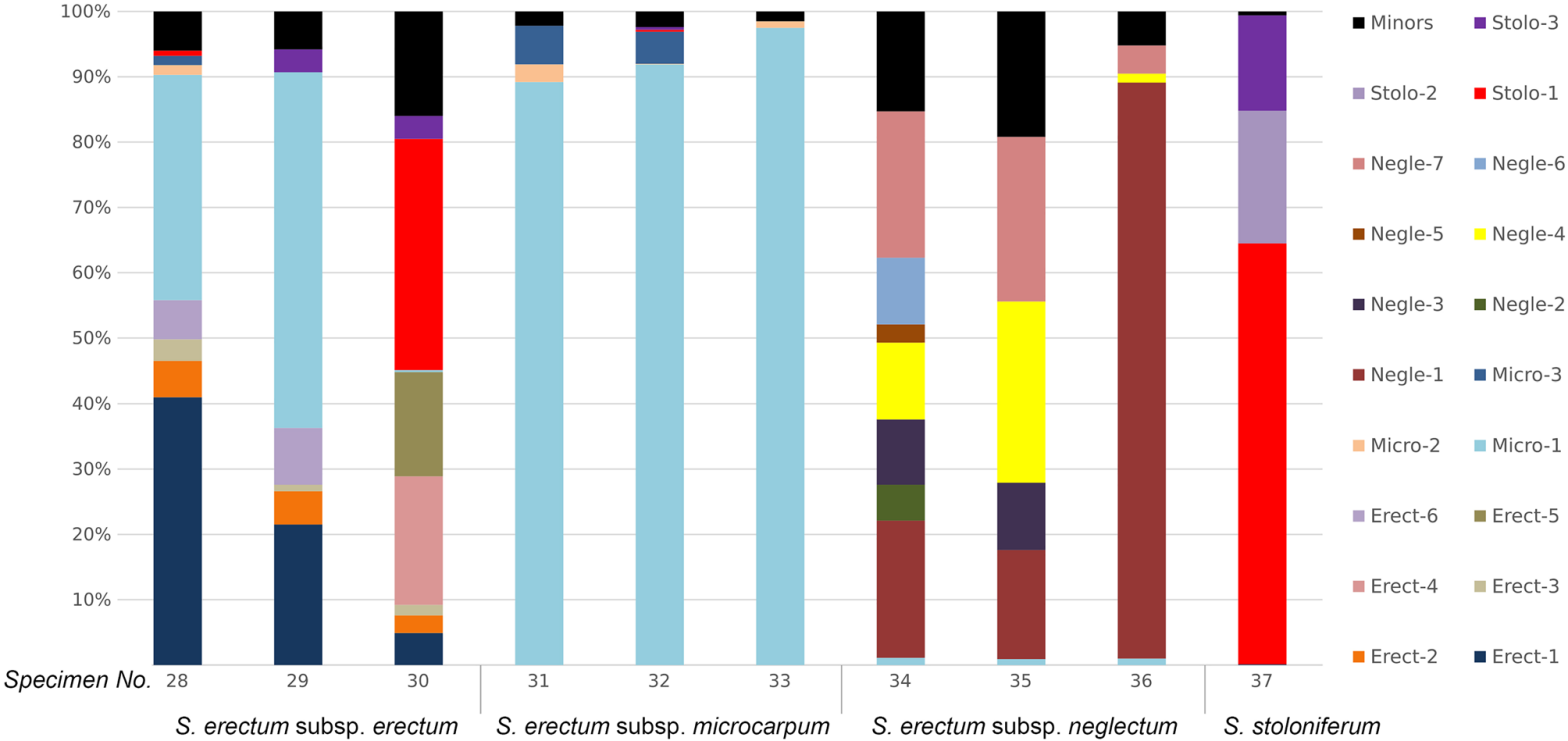
Stacked bar plots showing the relative abundance of the major ribotypes across the species of subgenus *Sparganium* section *Erecta*. Minor ribotypes in a sample are collectively termed "Minors".

The number of major ribotypes detected in the genomes of different species varies (Fig. 2, 3, 4). The species studied can be divided into three groups containing one, two, and more than two major ribotypes. Thus, *S. angustifolium* Michx., *S. fallax* Graebn., and *S. subglobosum* Morong in section *Natantia* of the subgenus *Xanthosparganium* (Fig. 2), have one major ribotype. Samples of *S. glomeratum* Laest. ex Beurl.) Beurl. have both one and two major ribotypes. Chinese samples of *S. emersum* (No. 4 and No. 5), *S. gramineum* Georgi, and *S. probatovae* No. 20 show two major ribotypes (Fig. 2). Samples of *S.* × *longifolium* Turcz. ex Ledeb., European *S. emersum* (No. 6), *S. probatovae* No. 21 and *S. rothertii*, have from three to five major ribotypes (Fig. 2). Samples of both species of *S. hyperboreum* Laest. ex Beurl. and *S. natans* L. in section *Minima* of the subgenus *Xanthosparganium* contain from two to six major ribotypes (Fig. 3). In section *Erecta* of the subgenus *Sparganium*, samples of *S. erectum* subsp. *erectum* L. have from five to seven major ribotypes, *S. erectum* subsp. *microcarpum* (Neuman) Domin from one to three, *S. erectum* subsp. *neglectum* (Beeby) Schinz & Thell. from two to seven, and *S. stoloniferum* subsp. *choui* (D. Yu) K. Sun contains three ribotypes (Fig. 4).

### Heterogeneity in ribotype composition

Many ribotypes are not species-specific and occur concurrently in a few species. For example, *S. glomeratum* from section *Natantia* has three ribotypes belonging to section *Minima*. Samples of *S.* × *longifolium* contain most of the ribotypes of *S. emersum* No. 6 and one minor ribotype of *S. gramineum*. *S. rothertii* has both *S. emersum* ribotypes No. 4 and No. 5, and *S. emersum* No. 6. Samples of *S. probatovae* have phylogenetically diverse ribotypes belonging to all three sections of the genus *Sparganium* (Fig. 2). Therefore, *S. probatovae* No. 20 contains ribotypes found in *S. emersum* / *S. rothertii*, the "Prob-1" ribotype of section *Minima*, which was not observed in other species, a minor number of *S. hyperboreum* / *S. natans* ribotypes from section *Minima*, and a minor number of rDNA of *S. erectum* subsp. *erectum* from section *Erecta*. *S. probatovae* No. 21 has the ribotypes of *S. gramineum*, *S. emersum* / *S. rothertii* and *S. erectum* s.l.

The species *S. emersum* and *S. probatovae* of section *Natantia* show considerable intraspecific differences between the samples in terms of rDNA composition. Therefore, sample *S. emersum* No. 6 from Yaroslavl Oblast, a region in the European part of Russia and samples *S. emersum* No. 4 and 5 from Inner Mongolia, China, do not have a common ribotype. Samples *S. probatovae* No. 20 from the Magadan Oblast and *S. probatovae* No. 21 from the Tyumen Oblast contain a total of six major ribotypes, with only two of them being common (Fig. 2).

Species of section *Minima* differ greatly from each other in rDNA composition and have only two common ribotypes (Fig. 3). Both species include rDNA ribotypes of section *Natantia* in their genome, probably originating from *S. emersum*, with *S. hyperboreum* also having ribotypes of *S. glomeratum*. Besides, *S. hyperboreum* No. 22 shows a minor quantity of *S. erectum* s.l. rDNA from section *Erecta*.

The subspecies of *S. erectum* differ in the set and abundance of ribotypes, with the most notable differences observed in *S. erectum* subsp. *neglectum*, which has only one ribotype in minor quantities shared with the other subspecies (Fig. 4). Furthermore *S. erectum* subsp. *microcarpum* has a more homogenized rDNA compared to other subspecies. It is worth noting that some samples of *S. erectum* contain rDNA variants of section *Natantia*. For example, *S. erectum* subsp. *erectum* has minor quantities of *S. emersum* rDNA, with *S. erectum* subsp. *neglectum* containing minor quantities of *S. glomeratum* rDNA. *S. stoloniferum* subsp. *choui*, in addition to species-specific ribotypes, carries ribotypes common to *S. eretum* subsp. *erectum* and *S. erectum* subsp. *microcarpum*.

## Discussion

### Organization and evolution of 35S rDNA in plants

Ribosomal 35S rRNA genes in plant genomes exist in a few thousands of copies that are reiterated tandemly at one or more loci which are visible as secondary constrictions or nuclear organizers (NORs) when transcriptionally active^26^. A noteworthy property of 35S rRNA genes is that the sequences of internal (ITS) and external (ETS) transcribed spacers are highly variable between species yet relatively homogeneous within the species genome and often species-specific^47,48^. This makes the comparative study of ITS and ETS sequences in different species a fruitful approach in the molecular phylogeny and DNA barcoding^47–49^. It is not well understood how the difference between the repeated 35S rRNA genes of sister species arises in the course of species divergence. This phenomenon is best consistent with the "birth-and-death" model of evolution of tandem repeats according to which one of the multiple copies of the rDNA locus with its typical set of SNPs is multiplied, while the other rDNA variants of this locus disappear^40,50,51^. The mechanisms for the rapid loss of many rDNA copies and the compensatory amplification of one of the copies are unknown. They are assumed to be in some way correlated with the emergence and repair of DNA double-strand breaks^52,53^, rolling replication of one of the chromosomal rDNA copies^54^ and/or extrachromosomal circular rDNA molecules^55^.

A logical consequence of rDNA homogenization in the plant genome is that only one type of rDNA sequences is detected in the genomes of many species by Sanger genomic sequencing, as observed in *Zingeria trichopoda* (Boiss.) P.A. Smirn.^56^, diploid and polyploid *Avena* L.^57^, some species of *Gossypium* L.^58^, and *Brassica* L.^59^. Generally, the phenomenon of nucleolar dominance is observed in hybrids and allopolyploids^47,60^. Here, the rDNA sequences of the repressed genome rapidly accumulate SNPs and deletions and are gradually lost^34,36,38,40,61^. However, a great many hybrids and species of hybrid origin show greater or lesser intragenomic heterogeneity of ITS^21,22,31,32^. There are a few reasons for the heterogeneity. The first is the retention of part of the rDNA ancestral species in hybrids^29–32^. The second reason could be pseudogenization of transcriptionally inert rDNA loci with their slow homogenization; it has been shown that in transcriptionally inert rDNA copies the accumulation rate of SNPs and deletions is 10 times higher than in rDNA of transcribed loci^38,40,61,62^. Homogenization of sequences within a single NOR proceeds faster than in different NORs, where it also occurs at varying rates^63,64^.

### Sparganium species with homogenized rDNA

We assumed that *Sparganium* species with a single major ribotype and low intragenomic polymorphism, such as *S. angustifolium, S. fallax* and *S. subglobosum*, might have diverged earlier than other representatives of section *Natantia*. This assumption is consistent with the earlier phylogenetic study based on cpDNA and Sanger rDNA sequences^2^. These species differ greatly from each other in the morphology of the generative sphere. The population system of the species typically consists of geographically or ecologically isolated or semi-isolated populations. It should be emphasized that populations of aquatic plants are considered to be more reproductively isolated in geographical and ecological terms as compared to those of terrestrial plants^15,65,66^. It is very likely that even if they are of hybrid origin, the process of rDNA homogenization in these plants is complete. It is worth mentioning that most minor variants of *S. fallax* prove to be phylogenetically close to the major one, except for a small number of distant variants that may testify to ancient hybridizations. Note that only one pair of Ag-NORs loci^67^ was revealed in the *S. fallax* genome. Unfortunately, this is the only species of the genus *Sparganium* with a known number of active NORs. Other species may have from one to two secondary constrictions, as we see in *Typha* species that are closely related to *Sparganium*^68^.

### rDNA composition and hybridization in different sections of the genus Sparganium

#### Section *Natantia*

The environmental or ecological isolation in the genus *Sparganium* is often due to the trophic status of water bodies where a particular species occurs. For example, *S. gramineum* inhabits oligotrophic and oligo-mesotrophic water bodies^1,69^, while *S. angustifolium* tends to dystrophic and oligotrophic ones^8,70,71^. Although the ranges of these species overlap, they generally do not share the same water body. *S. emersum* usually favors mesotrophic and eutrophic waters, though it can be found in the same water body as *S. angustifolium* and *S. gramineum*^1,8,69^. The latter indicates a wide ecological amplitude of this species. Adjacent habitats of certain species lead to the formation of potential hybrid zones. These zones often produce hybrids in their phenotype combining characters of parental species (*S.* × *engleratum Graebn.* (*S. angustifolium* × *S. emersum*)^2,12^ and *S.* × *longifolium* (*S. emersum* × *S. gramineum*))^16,17,19^.

The *S. emersum* samples No. 4 and No. 5 collected in China differed from the European *S. emersum* sample No. 6 by narrower leaves (with a poorly developed keel) and closely spaced male and widely spaced (often extra-axillary) female capitate inflorescences. The same characters are diagnostic for *S. rothertii*^13^. It should be noted that identification of the samples from China was handicapped by the fact that the plants had no above-water leaves (only those floating on the water surface).

Considering the morphological differences between *S. emersum* European and Baikal-Far East-China populations, Tsvelev^13^ suggested dividing *S.* cf. *emersum* into different species, *S. emersum* and *S. rothertii*^13^. *S. rothertii* was identified by Tsvelev from *S. emersum*^13^ on the basis of the morphological traits of leaves, anthers, styles, and fruits, as well as geographic criteria. However, *S. rothertii* is currently not a widely recognized species, and Chinese and Japanese researchers treat *S. rothertii* as *S. emersum*^72,73^.

Since the set of rDNA variants of the European *S. emersum* sample differs fundamentally from the Chinese samples, the latter can be regarded as a different or new cryptic species. Moreover, *S. rothertii* can be considered as a separate species since its rDNA composition is considerably different from *S. emersum*, containing ribotypes of both Chinese and European samples. It is worth mentioning that the ribotypes of the European *S. emersum* sample are also found in *S. hyperborem* and *S. natans* (section *Minima*), as well as in *S. erectum* subsp. *erectum* (section *Erecta*).

Another species with high intragenomic and intraspecific rDNA polymorphism is *S. probatovae*. This species was described by Tsvelev^13^. Earlier, collectors referred it to *S. hyperboreum* Laest., *S. minimum* Hill (= *S. natans*), *S. simplex* Huds (= *S. emersum*), or *S. friesii* Beurl. (= *S. gramineum*)^74^. According to Tsvelev's^13^ hypothesis, *S. probatovae* may have formed as a result of hybridization between *S. emersum* and *S. hyperboreum*. The hybrid origin of *S. probatovae* is proved by the absence of ripe fruits in the available herbarium specimens^13,74,75^. Other arguments in favor of the hybrid origin of the samples identified as *S. probatovae* are, as follows: 1) scattered habitats of the collected samples within the overlapping ranges of the supposed parental species^74,75^ and 2) deviation of the morphological characters towards a particular parental species^75^.

Six highly diverse ribotypes were found in two studied samples of this species that had similar morphological characters (sample No. 20 from the Magadan Oblast and sample No. 21 from the Tyumen Oblast). At the same time only two of the ribotypes are shared by the two samples. Therefore, these samples apparently have different origins.

*S. probatovae* sample No. 20 contains the ribotypes of *S. rothertii, S. hyperboreum, S. natans,* one unique ribotype ("Prob-1") assigned to section *Minima* on the basis of phylogenetic analysis, and one ribotype found in *S. erectum* subsp. *erectum*. Thus, this sample may also have evolved through a number of several rounds of hybridization that probably involved *S. rothertii*, one of the species of section *Minima*, as well as *S. erectum* subsp. *erectum*.

*S. probatovae* sample No. 21 contains the ribotypes of *S. gramineum, S. rothertii*, one unique ribotype ("Prob-2") and one ribotype dominating in *S. erectum* s.l. from section *Erecta* of *Sparganium* subgenus. Based on the data, we can assume that *S. probatovae* sample No. 21 emerged as a result of several rounds of hybridization involving *S. gramineum, S. rothertii* and *S. erectum* s.l.

Thus, the found rDNA diversity suggests a hybrid origin of *S. probatovae* samples, which is consistent with the morphological and geographical data.

Based on the set of ITS1 variants, *S. glomeratum*, containing ribotypes of *S. hyperboreum*, is presumably an intersectional hybrid. Morphological characters also indicate a possible hybrid origin of this species. *S. glomeratum* has an intermediate position (in terms of style length of the fruit) between section *Natantia* and section *Minima* (for example, *S. glomeratum* has a style length of 1.3 ± 0.2 mm, *S. fallax—*2.3 ± 0.05 mm, *S. hyperboreum*—0.3 ± 0.1 mm) and features closely spaced upper pistillate inflorescences often observed in section *Minima* representatives.

It was previously shown that *S.* ×*longifolium* combines the characters of the parental species, *S. emersum* and *S. gramineum*^17–19^. The hybridogenic nature of this species has now been proven by molecular methods^17^. According to our data, *S.* ×*longifolium* contains at least four ribotypes of *S. emersum* No. 6, including two major ones. At the same time, the rDNA of *S. gramineum* is contained in only one sample of *S.* ×*longifolium* in minor amounts. This may indicate that *S.* ×*longifolium* is an ancient hybrid, and the rDNA of the *S. gramineum* was repressed and later lost. Yu et al.^17^ showed that hybridization between *S. emersum* and *S. gramineum* is asymmetrical, and *S. gramineum* mainly acts as maternal species. Thus, we can assume that paternal rDNA is predominantly present in the samples studied.

Compared to its ancestors, *S.* ×*longifolium* has a greater ecological amplitude of occurrence, i.e., in the context of adaptation it is superior to the assumed parental species (based on our data). This allows it to occupy new habitats and sometimes even displace "parental" species from their native ones^19^. It is our observation that *S.* ×*longifolium* is generally sterile and propagates vegetatively. However, the presence of a small number of fertile populations of this hybrid^18,19^ appears to testify to the potential formation of backcrosses, with some of them being fertile.

#### Section *Minima*

Both species of section *Minima* show clear differences from each other in the set of ribotypes and are highly heterogeneous. Of all the ribotypes identified in this section, only two are common to both species. Thus, our data do not evidence hybridization within the section. We found the remarkable differences in the set of ribotypes between *S. hyperboreum* and *S. natans* as unforeseen, since earlier specimens with intermediate morphological characters occurred in nature in the areas where the ranges of *S. hyperboreum* (circumpolar hypoarctic species) and *S. natans* (circumpolar boreal species) overlapped^18,20,76,77^. We believed that the morphologically intermediate specimens might have resulted from introgressive hybridization^1,14,76^. Based on a very different set of rDNA in *S. hyperboreum* and *S. natans*, we developed a new hypothesis that plants of section *Minima* featuring a "mosaic" of species-specific traits may not be introgressive hybrids but rather a manifestation of Vavilov's law of homologous series in hereditary variability^78,79^.

It appears from the presence of ribotypes common to section *Natantia* that *S. hyperboreum* and *S. natans* are of hybrid origin, which involved species of this section. Some of the ribotypes are shared with *S. emersum* and *S. glomeratum. S. natans* has also two ribotypes phylogenetically related to the section *Natantia*, though not found in other species. They may have come from a species that was not included in our sampling. Furthermore *S. hyperboreum*, has a minor ribotype predominant in section *Erecta*, suggesting possible hybridization with representatives of subgenus *Sparganium*.

As follows from the above, reproductive barriers between some species of the subgenus *Xanthosparganium* are weakened, and when the ranges overlap, transfer of genetic material occurs.

#### Section *Erecta*

We studied *S. stoloniferum* subsp. *choui* and three subspecies of *S. erectum* from section *Erecta* of the subgenus *Sparganium*. These taxa cannot be differentiated by morphological criteria in vegetative and flowering state and may be distinguished only by ripe fruits^1,5,9^.

rDNA of the representatives of this section is not well homogenized. *S. erectum* subsp. *microcarpum* is the only exception, showing one to three major ribotypes across specimens. *S. erectum* subsp. *neglectum* differs greatly from the other two subspecies of *S. erectum* in the set of ribotypes, and share only one common ribotype. *S. erectum* subsp. *erectum* and *S. erectum* subsp. *microcarpum* are closer to *S. stoloniferum*, having several common ribotypes. Note that *S. erectum* subsp. *erectum* and *S. erectum* subsp. *neglectum* each have one minor ribotype from the section *Natantia*. The former probably inherited it from *S. emersum* and the latter from *S. glomeratum*. Minor quantities of rDNA of section *Natantia* in *S. erectum* s.l. may indicate ancient hybridization between the subgenera.

It is rather difficult to make any specific assumptions about possible hybrids and their parents from the set of ribotypes within this section. However, it should be noted that the presence of verified hybrid combinations (*S. erectum* subsp. *erectum*× *S. erectum* subsp. *microcarpum* and *S. erectum* subsp. *microcarpum*× *S. erectum* subsp. *neglectum*)^9^ may testify to possible hybridization of *S. erectum* subsp. *microcarpum* with other subspecies. Additionally, the presence of morphologically similar fruits in *S. stoloniferum* specimens from the Rostov, Volgograd, and Astrakhan oblasts (in our observations) may be evidence for the possible hybrid combination of *S. stoloniferum*× *S. erectum* subsp. *erectum* in the Astrakhan oblast.

Píšová and Fer^9^ concluded that the barriers of reproductive isolation between subspecies of *S. erectum*. have not yet been finally determined. However, according to our observations, all these subspecies often occupy different ecological niches in water reservoirs. For example, in the European part of Russia, *S. erectum* subsp. *microcarpum* is predominantly a cold-water (European-Eastern Siberian plurizonal) species occurring in small rivers, whereas *S. erectum* subsp. *erectum* (European-Siberian boreo-meridional species) preferably inhabits well-heated still and slowly flowing waters (water storage basins, ponds, etc.). *S. erectum* subsp. *neglectum* is a more southern (European-Pereasian-North African sub-oceanic) species found both in plain areas of cool southern rivers and streams and in well-heated marshy reservoirs. Differentiation of ecological niches leads to ecological isolation of these subspecies and can be an important factor of further speciation.

The existence of already proved hybrids^9^, as well as the presence of common ribotypes in the subspecies *S. erectum* and *S. stoloniferum* may testify to a more or less regular gene flow between these taxa. In our opinion, the differences in the set of ITS1 variants, different genome sizes and verified hybrid combinations, as well as ecological and geographic patterns of distribution, may serve as an argument for attributing *S. erectum* subspecies to the status of individual species.

#### Hybridization within and between subgenera

Some species contain in their genomes rDNA of several sections at once. In particular, within the subgenus *Xanthosparganium*, *S. glomeratum* and *S. probatovae* No. 20 from the section *Natantia* have ribotypes of section *Minima*. Whereas *S. hyperboreum* and *S. natans* have ribotypes of *S. emersum* from the section *Natantia*.

Two *Sparganium* subgenera also have common ribotypes. Thus, *S. probatovae* and *S. hyperboreum* from the subgenus *Xanthosparganium* contain minor amounts of *S. erectum* s.l. ribotypes from the subgenus *Sparganium*. On the other hand, some specimens of *S. erectum* s.l. contain minor amounts of rDNA of *S. emersum* and *S. glomeratum* from subgenus *Sparganium*.

Note that while some researchers doubted the possibility of the formation of hybrids between representatives of both subgenera (e.g., *S. emersum* × *S. erectum* s.l.)^10,80^, others did not deny it^6,11,81^.

## Conclusion

A comparative analysis of the intragenomic polymorphism of the 18S rDNA-ITS1-5.8S rDNA region in 15 *Sparganium* species and subspecies has showed that the taxa vary in the number and composition of ITS1 variants. Specimens of *S. probatovae* contain different sets of diverse ribotypes, therefore they can be considered as hybrids of different origin. The Chinese specimens of *S. emersum* have a principally different set of ribotypes compared to that of European and can be considered as a distinct or new cryptic species. *S. rothertii* differs from all *S. emersum* specimens, having ribotypes of both populations and so can be treated as a separate species. Furthermore we suggest that subspecies of *S. erectum* can be treated as separate species as well.

Species such as *S. angustifolium, S. fallax* and *S. subglobosum* have a homogenized rDNA bearing one major ribotype. Species with two major ribotypes include *S. gramineum* and Chinese specimens of *S. emersum*. Most taxa, however, contain three or more major ribotypes. Taxa with two or more major ribotypes are probably of hybrid origin. Their reproductive barriers may be weakened, particularly when the ranges of the taxa overlap. In most cases, hybridization is observed between the taxa at the subgeneric level. The fact that each subgenus contains a minor amount of rDNA from another subgenus implies the possibility of rare hybridization events between them.

## Methods

### Nomenclature and sampling

**Figure 5:**
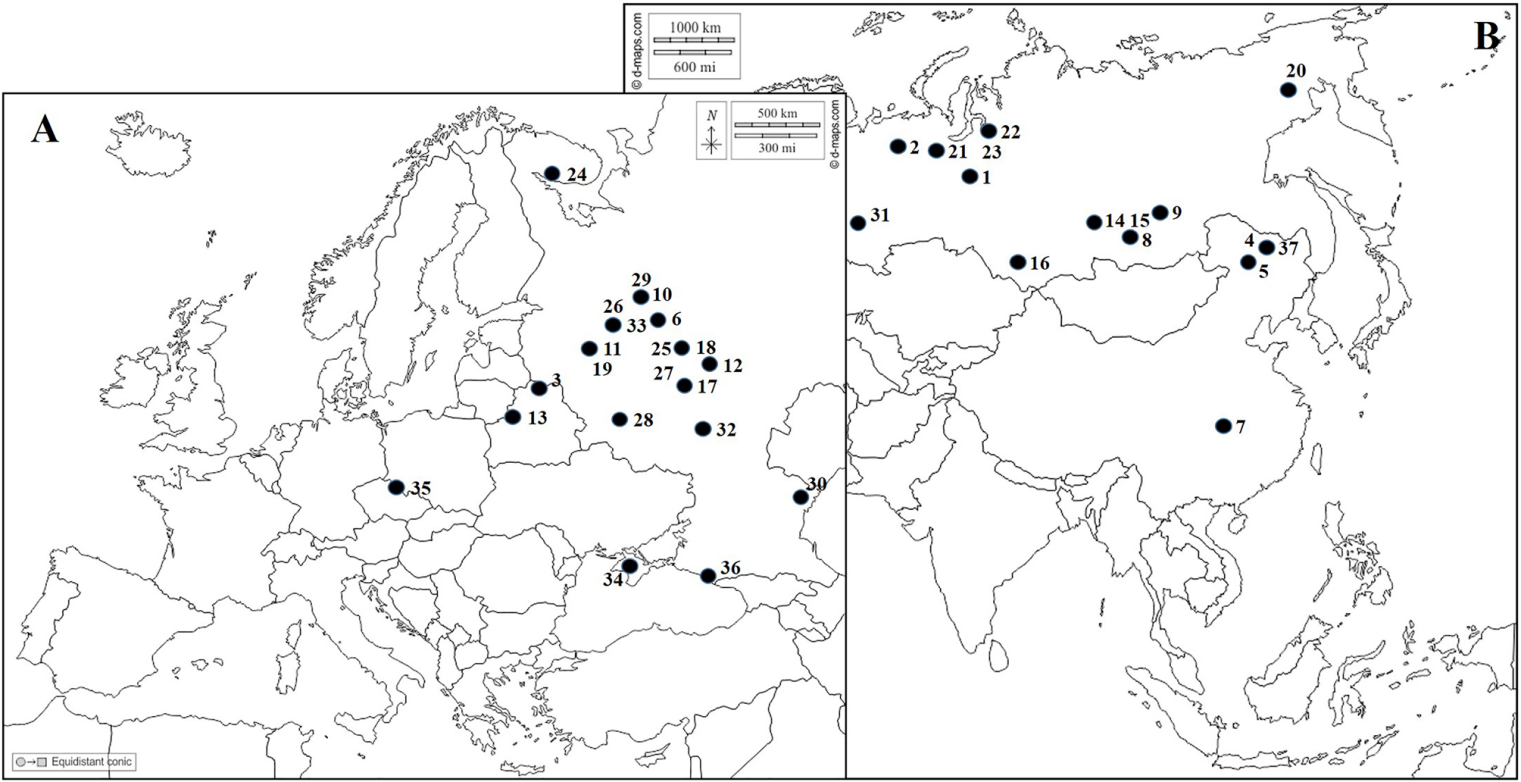
The Map showing location (black dots with numbers) of the *Sparganium* specimens analyzed in this study. Specimens are numbered as in Supplementary S3. (The basis of the maps were taken from d-maps.com (http://data.danetsoft.com/d-maps.com/) Europe (**A**) (https://d-maps.com/carte.php?num_car=2233&lang=en) and Asia (**B**) (https://d-maps.com/carte.php?num_car=55&lang=en), the image is changed using Free Software for Digital Photo Editing Paint.NET 4.3.11 https://paintnet.ru/download/).

We have studied a total of 37 specimens belonging to 15 species and subspecies of *Sparganium* collected in Eurasia (Supplementary Table S3, Fig. 5). Six specimens of two species were selected from the section *Minima*: *S. hyperboreum* Laest. ex Beurl., *S. natans* L. 21 specimens of 9 species were sampled from the section *Natantia*: *S. angustifolium* Michx., *S. emersum* Rehm., *S. fallax* Graebn., *S. glomeratum* (Laest. ex Beurl.) Beurl., *S. gramineum* Georgi, *S. probatovae* Tzvelev, *S. rothertii* Tzvelev, *S. subglobosum* Morong, S. × *longifolium* Turcz. ex Ledeb. 10 specimens of 4 species and subspecies were sampled from the section *Erecta*: *S. erectum* L. subsp. *erectum*, *S. erectum* subsp. *microcarpum* (Neuman) Domin, *S. erectum* subsp. *neglectum* (Beeby) K. Richt.), *S. stoloniferum* subsp. *choui* (D. Yu) K. Sun. Previously *S. stoloniferum* was treated as a subspecies *S. erectum* subsp. *stoloniferum* (Graebn.) H. Hara due to its fruit morphological features^11^. But we accept the *S. stoloniferum* subsp. *choui* as subspecies of *S. stoloniferum* based on the recent molecular data^82^. The herbarium specimens are stored in the Herbarium of Biological Faculty of the Moscow State University (MW), Herbarium of the Institute of Biology of Inland Waters RAS (IBIW), Skvortsov Herbarium of the Main Botanical Garden, Russian Academy of Sciences (MHA) and the personal collection of E.A. Belyakov (PCB). Information on collection locations, collectors, and other collection details of the samples studied is given in the Supplementary S3.

### DNA isolation, NGS sequencing and data analysis

DNA from herbarium specimens was extracted using the CTAB-method^83^. All specimens were divided into three parts and each part was processed independently (See Supplementary S4). ITS1 library preparation and pair-end sequencing on the Illumina MiSeq were performed at the Core Centre of “Genomic Technologies, Proteomics and Cell Biology” of the All-Russia Research Institute for Agricultural Microbiology (St. Petersburg, Russia) in turn for each of the three parts. The obtained reads were processed using the USEARCH 11.0 software^45^ according to the recommended protocol. In brief, all samples were pooled, pair-end reads were merged, trimmed and quality filtered according to the maximum expected error threshold of 1.0. Then reads were dereplicated and denoised by the UNOISE3 algorithm^46^ accompanied by the removal of chimeric sequences. Obtained ZOTUs with a size of at least 10 were blasted against GenBank *Sparganium* records to filter out off-target sequences and used to construct the ZOTU table. FASTA file containing the ZOTU sequences that were used to construct the ZOTU table is given in Supplementary S5. Table cells that had less than 15 read counts were nulled and the percentage of each ribotype in the total number of reads per specimen was calculated for all specimens. Ribotypes were divided into minor and major with a threshold value of 2%. Supplementary S6 contains FASTA file with the major ribotypes.

Thirty-two NCBI Sanger sequences of the genera *Typa* and *Sparganium* were added to the dataset (Supplementary S7). Sequence alignment was obtained by using MUSCLE^84^ and then checked manually in MegaX^85^. The maximum likelihood phylogenetic tree was built in IQ-TREE v.1.6.1^86^ under the best-fitted model with 1000 bootstrap replicates and visualized in iTOL^87^.

Experimental research and field studies on plants, including the collection of plant material, comply with relevant institutional, national, and international guidelines and legislation.

## Supplementary Information

Supplementary Information 1.

Supplementary Information 2.

Supplementary Information 3.

Supplementary Information 4.

Supplementary Information 5.

Supplementary Information 6.

Supplementary Information 7.

## Data Availability

Raw data are available at the NCBI Sequence Read Archive (SRA) under the BioProject PRJNA828568 BioSample accession numbers SAMN27672178–SAMN27672214.
