# Supplementary material for: Hybridization and diversity of aquatic macrophyte Sparganium L. (Typhaceae) as revealed by high-throughput nrDNA sequencing
Source: Sci Rep. 2022 Dec 14;12:21610. doi: 10.1038/s41598-022-25954-0 (PMC9750990; doi:10.1038/s41598-022-25954-0)

Supplementary Information

Hybridization and diversity of aquatic macrophyte *Sparganium* L. (Typhaceae) as revealed by high-throughput nrDNA sequencing

Evgeny A. Belyakov, Yulia V. Mikhaylova, Eduard M. Machs, Peter M. Zhurbenko & Aleksandr V. Rodionov

Authors for correspondence:

Evgeny A. Belyakov, Papanin Institute for Biology of Inland Waters Russian Academy of Sciences, Cherepovets State University, Russia.

E-mail: eugenybeliakov@yandex.ru

Yulia V. Mikhaylova, Komarov Botanical Institute of the Russian Academy of Sciences, Russia.

E-mail: YMikhaylova@binran.ru

Table S2. The nucleotide substitutions in the main ribotypes of representatives of the genus *Sparganium*

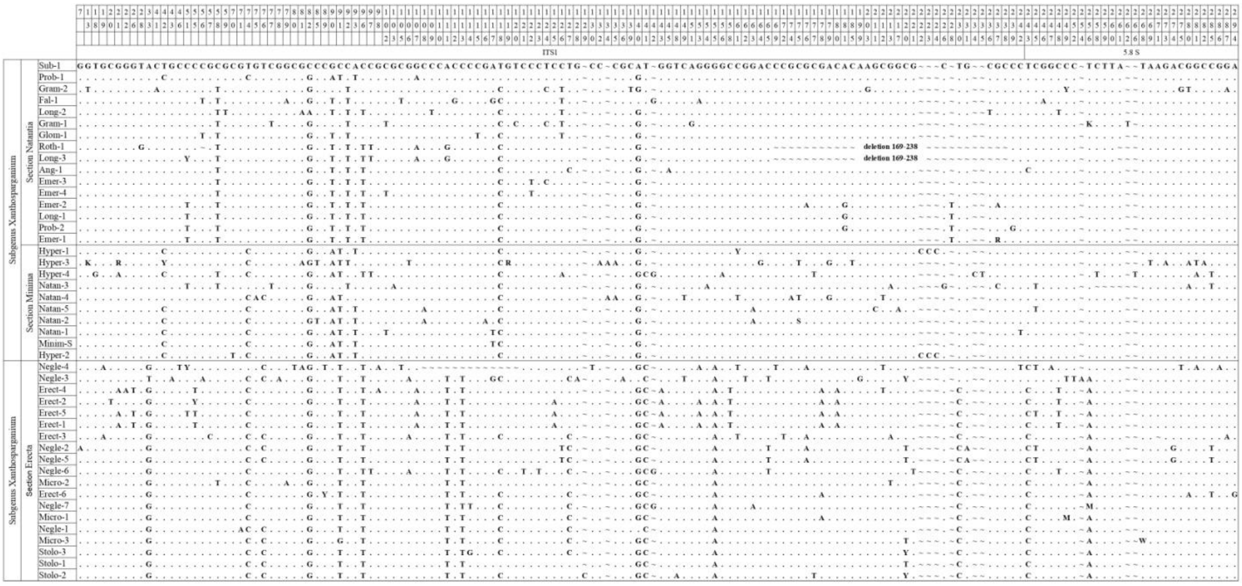

Supplement: Supplementary file 2 — Supplementary Information 2. [file 41598_2022_25954_MOESM2_ESM.pdf]
