# Supplementary material for: Hybridization and diversity of aquatic macrophyte Sparganium L. (Typhaceae) as revealed by high-throughput nrDNA sequencing
Source: Sci Rep. 2022 Dec 14;12:21610. doi: 10.1038/s41598-022-25954-0 (PMC9750990; doi:10.1038/s41598-022-25954-0)
Supplement: Supplementary file 3 — Supplementary Information 3. [file 41598_2022_25954_MOESM3_ESM.pdf]

Supplementary Information

Hybridization and diversity of aquatic macrophyte *Sparganium* L. (Typhaceae) as revealed by high-throughput nrDNA sequencing

Evgeny A. Belyakov, Yulia V. Mikhaylova, Eduard M. Machs, Peter M. Zhurbenko & Aleksandr V. Rodionov

Authors for correspondence:

Evgeny A. Belyakov, Papanin Institute for Biology of Inland Waters Russian Academy of Sciences, Cherepovets State University, Russia.

E-mail: eugenybeliakov@yandex.ru

Yulia V. Mikhaylova, Komarov Botanical Institute of the Russian Academy of Sciences, Russia.

E-mail: YMikhaylova@binran.ru

Supplementary S3. The List of taxa and vouchers for the material examined.

Subgenus *Hanthosparganium*. Section *Natantia* Aschers. et. Graebner.: *Sparganium angustifolium* Michx.: *1.* – Russia, Khanty-Mansi Autonomous Area, Surgutsky Distr., Fyodorovskoye Field, a water-flooded oil sludge pit, coll. and det. by Ye.A. Shishkonakova, N.K. Shvedchikova, 20 VIII 2013, MW; ***2.*** – the Komi Republic, Ukhtinsky Distr., Yarega Settl., an oil mine's water storage reservoir, No. 1 west of the dam, coll. and det. by B.Yu. Teteryuk, 04 VIII 2015, E.A. Belyakov's personal collection (BPC); ***3.*** – Pskov Region, Sebezhsky Distr., the vicinity of Sebezhsky National Park, Lake Maly Zelenets, coll. and det.

by E.A. Belyakov, 30 VIII 2019, BPC; ***S. emersum* Rehm.: *4.*** – China, the Inner Mongolia, Hulunbuir, Arun Banner, the Allen River, 48.669163, 122.782546, coll. and det. by E.A. Belyakov, 12 VIII 2019, fresh leaves were sampled and dried in silica gel for subsequent DNA extraction; ***5.*** – China, the Inner Mongolia, Hulunbuir, Arun Banner, Xinfachaoxian Town, the Alun River, 48.144722, 123.416944, coll. and det. by E.A. Belyakov, 10 VIII 2019, fresh leaves were sampled and dried in silica gel for subsequent DNA extraction; ***6.*** – Russia, the Yaroslavl Region, Rostovsky Distr., the vicinity of Zaozyorye Vill., Lake Zaozyorye, coll. and det. by Xinwei Xu, 08.07.2018, BPC; ***S. fallax* Graebn.: *7.*** – China, Shénnóngjià Forestry District, small lakes in a wetland in a mountain basin, 31.48139262, 110.00850868, coll. and det. by E.A. Belyakov, 03 VIII 2019, fresh leaves were sampled and dried in silica gel for subsequent DNA extraction; ***S. glomeratum* (Laest. ex Beurl.) Beurl.: *8.*** – Russia, Irkutsk Region., Ziminsky Distr., Osipovsky Settl., a flowing dead arm of Oka River throughout the settlement's territory, in shallow water, coll. and det. by V. Chepinoga, T. Chulitskaya, 09 VII 2005, IBIW; ***9.*** – the Republic of Buryatia, Severo-Baikalsky Distr., Barguzin Biosphere Reserve, bay coast near the Bolshaya Rechka River, bog lakes behind the pebblestone spit of Lake Baikal, on a mass scale, coll. by V.S. Vishnyakov, 02 IX 2019, det. by E.A. Belyakov, BPC; ***10.*** – the Yaroslavl Region, Poshekhonsky Distr., the vicinity of Golodyaika Vill., in a forest ditch, coll. and det. by E.A. Belyakov, 10 IX 2016, BPC; ***S. gramineum* Georgi: *11.*** – Russia, Tver Region, Ostashkovsky Distr., Lake Sabro in the vicinity of Zvyagino Vill., the eastern shore, coll. by E.A. Belyakov, A.G. Lapirov, 10 VII 2014, det. by E.A. Belyakov, BPC; ***12.*** – Nizhny Novgorod Region, Vorotynsky Distr., Lake Maloye Plotovo, in the vicinity of Kuzmiyar Settl., coll. and det. by E.A. Belyakov et al., 04 IX 2018, BPC; ***13.*** – the Republic of Belarus, Minsk Region, Myadelsky Distr., ~1 km southwest of Shishkovichi Vill., Lake Zaportovo shore, to the right of Myadel-Vileyka highway, in the water, at a depth of about 1 m, coll. and det. by M. Jus, 04 IX 2018, MW; ***S.***

probatovae **Tzvelev:** ***20.*** – Russia, Magadan Region, Srednekansky Distr., Seymchan, a pier, an arm in the Kolyma River flood plain, coll. by Berkutenko, 29 VII 1974, det. by A.V. Grebenjuk, MHA; ***21.*** – the Tyumen Region, Berezovsky Distr., 15 km east of Beloyarsky Settl., along the Beloyarsky-Urengoy route, lake shore, sand, coll. by Ye.Ye. Timoshok, Ye.G. Naumova, Ye. Senchenko, S. Schelokova, 22 VII 1988, det. by A.V. Grebenjuk, MW; ***S. rothertii*** **Tzvelev:** ***14.*** – Russia, Irkutsk Region, Ziminsky Distr., the vicinity of Zulumay Settl., in the right-bank dead arm of Zima River, coll. by V.V. Chepinoga, S. Rosbakh, 12 VII 2005, det. by V.V. Chepinoga, IBIW; ***15.*** – Irkutsk Region, Irkutsky Distr., Urik Vill., dead arm of Kuda River, coll. by V.S. Vishnyakov, 11 VII 2018, det. by E.A. Belyakov, BPC; ***S. subglobosum*** **Morong:** ***16.*** – Russia, the Altai Republic, Mayminsky Distr., near Ozyornoye Vill., Lake Manzherok, coll. by Ye.Yu. Zarubina, 09 VII 2010, det. by E.A. Belyakov, IBIW; ***S.×longifolium*** **Turcz. ex Ledeb.:** ***17.*** – Russia, Nizhny Novgorod Region, Dzerzhinsk City Distr., the vicinity of Pyra Settl., Lake Pyrskoye, coll. and det. by E.A. Belyakov, 07 IX 2018, BPC; ***18.*** – Ivanovo Region, Savinsky Distr., in the vicinity of Goryachevo Vill., Lake Polyovo, coll. and det. by E.A. Belyakov, 01 IX 2018, BPC; ***19.*** – Tver Region., Ostashkovsky Distr., Lake Sabro, coll. and det. by E.A. Belyakov, 09 VIII 2016, BPC; **Section *Minima* Aschers. et. Graebner.:** ***S. hyperboreum*** **Laest. ex Beurl.:** ***22.*** – Russia, the Tyumen Region, Tazovsky Distr., the vicinity of Tazovsky Settl., in a flooded roadside hollow, coll. and det. by S.A. Nikolayenko, 01 VIII 2015, BPC; ***23.*** – the same habitat, a sprout grown from a fruitlet in the laboratory; ***24.*** – Murmansk Region, Tersky District, the White Sea, Porya Guba bay, Lake Ozerchanka, the east. part of the isl., in a flooded rock basin, coll. and det. by M.N. Kozhin, 11 VIII 2006, MW; ***S. natans*** **L.:** ***25.*** – Russia, the Yaroslavl Region, Rostovsky Distr., a sand pit in the vicinity of Chashnitskoye Vill., in a drain ditch, in the water, coll. and det. by E.A. Belyakov, 30 VII 2016, BPC; ***26.*** – Tver Region, Udomelsky Distr., a small swamp in the vicinity of Lake Kezadra, coll. and det. by

E.A. Belyakov, 14 VIII 2018, BPC; ***27.*** – Nizhny Novgorod Region, Dzerzhinsk City Distr., the vicinity of Pyra Settl., a former peat mine in the vicinity of Lake Pyrskoye, coll. and det. by E.A. Belyakov et al., 08 IX 2018, BPC; **Subgenus *Sparganium*. Section *Erecta* Aschers. et. Graebner.: *S. erectum* subsp. *erectum* L.: *28.*** – Russia, Kaluga Region, Kaluga, Oka River, coll. and det. by E.A. Belyakov, 09 VIII 2013, IBIW; ***29.*** – Yaroslavl Region, Poshekhonsky Distr., the vicinity of Malafeyevo Vill., Rybinskoye water storage reservoir’s shore, in the baymouth of Matkoma River, coll. and det. by E.A. Belyakov, 15 IX 2015, IBIW; ***30.*** – Astrakhan Region, Volodarsky Distr., the vicinity of Aktyube Vill., water meadow at the m/way, 46.408611, 48.401389, coll. and det. by E.A. Belyakov, 27 VII 2018, BPC; ***S. erectum* L. subsp. *microcarpum* (Neuman) Domin: *31.*** – Russia, the Republic of Udmurtia, Izhevsk, Lineiny Lane, in a dried roadside hollow, coll. by E.A. Belyakov, O.A. Kapitonova, 23 VII 2013, det. by E.A. Belyakov, IBIW; ***32.*** –Tambov Region, Morshansky Distr., the vicinity of Molodyozhny Settl., Serp River, 53.651133, 41.715370, coll. and det. by E.A. Belyakov, 26 VII 2019, BPC; ***33.*** – Russia, Tver Region, Andreapolsky Distr., the vicinity of Bologovo Vill., Lake Parshinskoye, 56°54`27,59`` 31°42`19,84``, coll. by E.A. Belyakov, E.V. Garin, 14 VIII 2016, det. by E.A. Belyakov, BPC; ***S. erectum* L. subsp. *neglectum* (Beeby) Schinz & Thell.: *34.*** – the Republic of Crimea, Yalta City Distr., the southern side of the main Crimean mountain range, at the beginning of the 10th km of Yalta-Bakhchisaray highway, the Kara-Gol shrinking forest lake, coll. and det. by V.V. Markov, 15 X 2008, MHA; ***35.*** – Slask Dolny, Rakowice Wielkie, Lwówek Sĺaski, Lower Silesian Voivodeship, the coast of the oxbow in the valley of the Bobr River, S-E of the Lower Silesia Village, on the shore of the Lake in the valley of the Bobr River, from the Village to the S-E, coll. by E. Kozioł, H. Charytonowicz, 23.VIII.2008, det. by A.V. Grebenjuk, MHA; ***36.*** – Russia, the Republic of Adygea, Maikopsky Distr., the vicinity of Krasnooktyabrsky Settl. (near Maikop), in a small swamp, coll. and det. by E.A. Belyakov, 24 VII 2019, BPC; ***S.***

stoloniferum* subsp. *choui* (D. Yu) K. Sun:** ***37. – China, Heilongjiang Province, Heihe City District, in the fishery ponds, coll. and det. by E.A. Belyakov, 08 VIII 2019, fresh leaves were sampled and dried in silica gel for subsequent DNA extraction.
