# Supplementary material for: Hybridization and diversity of aquatic macrophyte Sparganium L. (Typhaceae) as revealed by high-throughput nrDNA sequencing
Source: Sci Rep. 2022 Dec 14;12:21610. doi: 10.1038/s41598-022-25954-0 (PMC9750990; doi:10.1038/s41598-022-25954-0)
Supplement: Supplementary file 4 — Supplementary Information 4. [file 41598_2022_25954_MOESM4_ESM.pdf]

Supplementary Information

Hybridization and diversity of aquatic macrophyte *Sparganium* L. (Typhaceae) as revealed by high-throughput nrDNA sequencing

Evgeny A. Belyakov, Yulia V. Mikhaylova, Eduard M. Machs, Peter M. Zhurbenko & Aleksandr V. Rodionov

Authors for correspondence:

Evgeny A. Belyakov, Papanin Institute for Biology of Inland Waters Russian Academy of Sciences, Cherepovets State University, Russia.

E-mail: eugenybeliakov@yandex.ru

Yulia V. Mikhaylova, Komarov Botanical Institute of the Russian Academy of Sciences, Russia.

E-mail: YMikhaylova@binran.ru

Supplementary S4. Sample separation

All specimens were divided into three parts and each part was processed independently, including library preparation and Illumina MiSeq sequencing. Each part is marked with a different letter A, X and E.

Sparganuim_9 X
Sparganuim_8 A
Sparganuim_6 A
Sparganuim_5 X
Sparganuim_37 X
Sparganuim_36 X
Sparganuim_35 A
Sparganuim_34 A
Sparganuim_33 A
Sparganuim_32 X
Sparganuim_31 A
Sparganuim_4 X

Sparganuim_30 A
Sparganuim_29 A
Sparganuim_28 A
Sparganuim_27 A
Sparganuim_26 A
Sparganuim_24 A
Sparganuim_23 E
Sparganuim_22 E
Sparganuim_21 X
Sparganuim_3 X
Sparganuim_20 A
Sparganuim_18 A
Sparganuim_17 A
Sparganuim_16 X
Sparganuim_15 A
Sparganuim_14 A
Sparganuim_13 A
Sparganuim_12 A
Sparganuim_11 A
Sparganuim_1 A
Sparganuim_10 E
Sparganuim_19 A
Sparganuim_2 A
Sparganuim_25 A
Sparganuim_7 X
