# Supplementary material for: Hybridization and diversity of aquatic macrophyte Sparganium L. (Typhaceae) as revealed by high-throughput nrDNA sequencing
Source: Sci Rep. 2022 Dec 14;12:21610. doi: 10.1038/s41598-022-25954-0 (PMC9750990; doi:10.1038/s41598-022-25954-0)
Supplement: Supplementary file 7 — Supplementary Information 7. [file 41598_2022_25954_MOESM7_ESM.pdf]

Supplementary Information

Interspecific hybridization in the evolution of aquatic macrophyte bur-reed (*Sparganium* L., Typhaceae): insights from high-throughput sequencing of nrDNA

Evgeny A. Belyakov, Yulia V. Mikhaylova, Eduard M. Machs, Peter M. Zhurbenko & Aleksandr V. Rodionov

Authors for correspondence:

Evgeny A. Belyakov, Papanin Institute for Biology of Inland Waters Russian Academy of Sciences, Cherepovets State University, Russia.

E-mail: eugenybeliakov@yandex.ru

Yulia V. Mikhaylova, Komarov Botanical Institute of the Russian Academy of Sciences, Russia.

E-mail: YMikhaylova@binran.ru

Supplementary S7. The GenBank accession number used in this study

**KF265388**	***Typha angustifolia***
**EU924346**	***Typha domingensis***
**KF265389**	***Typha domingensis***
**KF265390**	***Typha latifolia***
**KF265368**	***Sparganium angustifolium***
**KF265370**	***Sparganium angustifolium***
**KF265369**	***Sparganium angustifolium***
**KF265391**	***Sparganium emersum***
**KF265392**	***Sparganium emersum***

**KF265371**	***Sparganium emersum***
**KF265393**	***Sparganium emersum***
**LC577742**	***Sparganium erectum***
**LC577749**	***Sparganium erectum***
**KF265394**	***Sparganium erectum subsp. microcarpum***
**LC577743**	***Sparganium fallax***
**LC577746**	***Sparganium fallax***
**LC577750**	***Sparganium fallax***
**LC577747**	***Sparganium fallax***
**KF265377**	***Sparganium fallax***
**KF265376**	***Sparganium fallax***
**KF265379**	***Sparganium glomeratum***
**KF265386**	***Sparganium glomeratum***
**KF265380**	***Sparganium glomeratum***
**KF265381**	***Sparganium gramineum***
**KF265383**	***Sparganium hyperboreum***
**KF265396**	***Sparganium hyperboreum***
**KF265385**	***Sparganium natans***
**KF265395**	***Sparganium stoloniferum***
**KF265373**	***Sparganium stoloniferum***
**KF265387**	***Sparganium subglobosum***
**LC577745**	***Sparganium subglobosum***
**KF265397**	***Sparganium subglobosum***
